# Cardiac arrest following cannabis use: a case report

**DOI:** 10.1186/1757-1626-2-208

**Published:** 2009-11-19

**Authors:** Abdo H Sattout, Mark F Nicol

**Affiliations:** 1 Department of Emergency Medicine, Macclesfield District General Hospital, Victoria Road, Macclesfield, Cheshire, SK10 3BL, UK

## Abstract

**Background:**

Cannabis, or *Marijuana*, remains one of the most universally used recreational drugs. Over the last four decades, its popularity has risen considerably as it became easily accessible and relatively affordable. Peak use is amongst the young aged 18 to 25 years, although these figures are now shifting towards earlier teens. A strongly installed culture still regards cannabis a harmless drug, yet as more reports have shown there are considerable adverse cardiovascular events linked with its use.

**Case Presentation:**

In this paper, we present the case of a 15-year-old male who suffered a cardiac arrest following cannabis use and survived the episode.

**Conclusion:**

Cardiac arrest is a rare and possibly fatal consequence of cannabis use. Public awareness should be raised by extensively promoting all potential complications associated with its use.

## Introduction

The recreational use of cannabis, or *marijuana*, has been on the rise since the 1960s. Its popularity has been catalysed by an easy accessibility and affordability. Peak use is generally amongst the young aged 18 to 25 years [1-3], although these figures are now shifting towards earlier teens [2]. Nevertheless, a strongly installed culture still regards cannabis harmless when compared to other existing illicit drugs [1]. As more reports have shown, there are considerable adverse cardiovascular events linked with its use.

## Case Report

A 15-year-old English Caucasian male was brought to our emergency department having suffered a witnessed cardiac arrest, following the use of alcohol and cannabis with friends. Paramedics attended the scene within three minutes of call being passed: cardiopulmonary resuscitation (CPR) was in progress by the patient's friends, and the first recorded rhythm was asystole. After two minutes of CPR, the rhythm changed into ventricular fibrillation (VF) which was successfully cardioverted into a sinus rhythm with a single DC shock (200 J).

On arrival to hospital, the patient was maintaining his airway with respiratory arrhythmia. His vital signs showed a blood pressure (BP) of 115/86, a heart rate (HR) of 86 bpm and a recorded GCS of 4 out of 15. In view of the respiratory arrhythmia and impaired GCS, he was intubated and ventilated.

Investigations conducted in the emergency department showed the initial 12-lead electrocardiogram (ECG) to be in a normal sinus rhythm without any acute ischaemia. No changes were identified on subsequent 12-lead ECGs. Urine drug screen (Princeton BioMeditech Corp., AccuSign^® ^DOA 4) was positive for THC, but negative for opiates, amphetamines and cocaine. Blood tests including haematology, biochemistry and gases were all within normal.

Radiological imaging revealed normal chest X-ray and computed tomography (CT) scan of the head. Of note, the patient had an episode of significant bradycardia whilst being scanned that necessitated a dose of intravenous Atropine.

Further information obtained from social workers disclosed the patient to suffer from attention deficit disorder (ADHD) along with a history of previous cocaine and ecstasy abuse. Although Ritalin was prescribed for his ADHD, he had failed to comply with the treatment in the last 18 months.

Our patient was admitted to a paediatric intensive care unit (PICU) at a neighbouring hospital. He was successfully extubated within 24 hours, and discharged from hospital 5 days later with no neurological or cardiovascular sequelae. Although arrangements were made for an outpatient follow up, he failed to attend his appointments and further cardiological assessments (such as echocardiography) never materialised.

## Discussion

*Cannabis sativa *or *marijuana*, is a plant that has been known to humankind as early as the 8^th ^century BC in ancient Mesopotamia [1]. It was purposely grown for its fibres that went into the production of rope and tissues, whereas its resin was regarded as a medication to alleviate pain and insomnia. Cannabis was brought to Europe in the 19^th ^century by the returning Napoleonic soldiers from the Middle East [4].

The resin holds more than 400 chemical compounds and 60 psychoactive materials known as cannabinoids with the most potent agent being delta-9-tetrahydrocannabinol (Δ^9^-THC, or simply THC) [1-5]. Progress made in agricultural techniques altered the THC content and as a result, the drug's potency was significantly enhanced. A joint in the 1960's would have averaged 10 mg of THC compared to 150-400 mg nowadays [2,5].

Cannabinoids, including THC, are exceptionally lipid soluble; tissue distribution is proportional to blood flow with peak accumulation in fatty tissues attained in 4 to 5 days. The plasma and tissue half-lives are around 30 hours and 7 days respectively [2,6]. It can thus be detected in urine samples several days after a single use or up to months in chronic abusers [6].

In humans, the biological effects of cannabis are mediated by two G-protein coupled receptors: CB_1 _and CB_2_. Both have been recognized to be cannabinoid specific. The CB_1 _receptors, known as neuronal cannabinoid receptors, are present mainly in the central and peripheral nervous system, but can also be found in the lungs, kidneys and liver. Activation of CB_1 _receptors inhibits the sympathetic response causing vasodilation that consequently leads to hypotension and bradycardia; an effect that is mediated by the endogenous cannabinoid neurotransmitters anandamide (*N*-arachidonoylethanolamine) and 2-AG (2-arachidonoylglycerol). CB_2 _receptors are present throughout most of the immune system and their activation might be implicated in nociception [1,2,5,7-9].

Cardiovascular manifestations of cannabis result from a biphasic dose-dependent physiological effect on the autonomic nervous system: low to moderate doses tend to cause tachycardia and raised blood pressure (proportional to an increase in cardiac output) by increasing the sympathetic activity, whilst high doses produce bradycardia and hypotension by increasing the parasympathetic activity [1-3,5,6,8]. Myocardial tissue ischaemia secondary to cannabis use is thought to be resulting from one of the following pathophysiological factors: (1) an increase in O_2 _demand secondary to increased heart rate, (2) a decreased O_2 _supply secondary to a rise in carboxyhaemoglobin levels post smoking or (3) a reduced blood flow secondary to arterial vasospasm.

Reported complication associated with cannabis use is myocardial infarction. It is assumed to be an outcome of myocardial tissue ischemia in people with either normal or atherosclerotic coronary arteries, with the risk of infarction increasing to almost 5-fold in the first hour amongst users [1,3,6,7]. To date, seven cases of myocardial infarction associated with cannabis use have been reported: six were male, age ranging from 17 to 48 years (a median of 32 years), and four had normal coronary vasculature [1,7].

Bachs and Morland [10] discussed six cases of acute cardiovascular death after cannabis use. The association was confirmed by a positive serum THC toxicology at post-mortem: all were male aged 17 to 43 years (a median of 40 years), and four had atheromatous coronary vasculature. Although similar fatal cases were also reported, the majority lacked toxicological evidence [3,7,10].

Cannabis, in conjunction with alcohol and drugs such as cocaine, creates a more pronounced synergetic effect on the myocardium: it triggers a significant tachycardia that leads to ischemia, and subsequently infarction and even death [3,6,11]. Finally, other reported cardiovascular complications included ventricular tachy-arrhythmias [3] and atrial fibrillation [3,5,7,8].

In our case, two facts need to be addressed. Firstly, the cannabis causality was assumed on the result of the positive urine drug screen; hence, a full toxicology screen was not pursued. Secondly, an inpatient echocardiograph was also not documented and later not performed as the patient failed to attend his outpatient's follow up. We feel the effects of chronic cannabis abuse were most likely potentiated by the alcohol consumption. This could have led to a sudden over-parasympathetic activity along with a possible tissue ischaemia, thus triggering the asystolic episode.

It is of interest to point out that there has been no report yet in the literature stating an asystolic cardiac arrest following cannabis use and survival at a young age.

## Conclusion

Recreational use of cannabis will remain on the rise with a number of potentially serious, if not fatal, consequences. Availability and affordability has resulted in it being not only accessible to an adult population, but also teenagers. Since 2004, cannabis in the UK has been classified as a 'class C' drug: a fact that is definitely carrying an immense health implication secondary to its consumption with calls already being raised to re-classify it as a 'class B'. Public awareness should be extensively made regarding all possible complications, and emergency physicians should not assume asystolic arrest has an inevitable fatal outcome.

## Abbreviations

THC: delta-9-tetrahydrocannabinol.

## Consent

Written informed consent was obtained from the patient's parents for publication of this case report. A copy of the written consent is available for review by the Editor-in-Chief of this journal.

## Competing interests

The authors declare that they have no competing interests.

## Authors' contributions

AS, was responsible for the case review, literature review and final manuscript drafting.

MN, was responsible for the manuscript critique and review.

All authors have read and approved the final manuscript.
